# Editorial: Accelerated Translation Using Microphysiological Organoid and Microfluidic Chip Models

**DOI:** 10.3389/fphar.2021.827172

**Published:** 2022-01-03

**Authors:** Kambez H. Benam, Janette K. Burgess, Alastair G. Stewart

**Affiliations:** ^1^ Division of Pulmonary, Allergy and Critical Care Medicine, Department of Medicine, University of Pittsburgh, Pittsburgh, PA, United States; ^2^ Department of Bioengineering, University of Pittsburgh, Pittsburgh, PA, United States; ^3^ Vascular Medicine Institute, University of Pittsburgh, Pittsburgh, PA, United States; ^4^ University of Groningen, University Medical Center Groningen, Department of Pathology and Medical Biology, Groningen, Netherlands; ^5^ University of Groningen, University Medical Center Groningen, Groningen Research Institute for Asthma and COPD (GRIAC), Groningen, Netherlands; ^6^ University of Groningen, University Medical Center Groningen, W.J. Kolff Institute for Biomedical Engineering and Materials Science, Groningen, Netherlands; ^7^ Department of Biochemistry and Pharmacology, School of Biomedical Science, University of Melbourne, Parkville, VIC, Australia; ^8^ ARC Centre for Personalized Therapeutics Technologies, University of Melbourne, Parkville, VIC, Australia

**Keywords:** microphysiological systems, organs-on-chips, organoids, predictive modeling, pharmacology, safety, efficacy, translational science

The discovery of new therapeutic modalities is a lengthy and costly process (Niemeyer et al., 2018). Despite considerable advances over the past few decades in our understanding of human pathophysiology and emergence of new assays and experimental toolkits, high failure rate in phase II and phase III clinical trials due to lack of compound efficacy remains a major challenge for the biopharmaceutical industry (Harrison, 2016). This, for most part, can be explained by limitations of preclinical models in predicting such failures early during drug development processes (Krishnan et al., 2016; Li and Stewart, 2021). As such, development, validation, and application of preclinical microphysiological systems (MPS), such as Organ-on-Chips and organoids, presents as an indubitable approach to tackle this pressing unmet need.

Organs-on-Chips are living microfluidic devices that are fabricated using computer microchip manufacturing methods and are commonly thumb-sized (Benam et al., 2015). They contain continuously or intermittently perfused microchannels inhabited by living cells or microtissues (Benam and Ingber, 2016; Benam et al., 2019). These platforms can recreate aspects of the physicochemical and biomechanical microenvironments of human organs, multicellular architecture, and inter-cellular as well as tissue–tissue crosstalk *in vitro* (Fustin et al., 2019). Organoids offer an alternative approach to recreate certain complexities of human organs *in vitro*. These are culture systems in which organ-specific progenitor cells can rapidly expand and generate multi-cellular 3D structures (Niemeyer et al., 2018). Organs-on-Chips and organoids, despite their reductionist nature and limitations (beyond the scope of this Editorial), are emerging technologies with substantial potential to improve predictive pharmacology in the preclinical space and to potentially reduce animal use, particularly where little supporting scientific evidence is present for the utility of the animal models in a particular condition.

The present research topic provides a brief, yet timely, snapshot of recent developments by pioneers in the MPS world on application of these platforms to enable more accurate human-relevant biology modeling for translational applications. The five articles in this Issue can be broadly categorized into three groups based on the organ systems on which they primarily focused: musculoskeletal system (Charrez et al.; Lee-Montiel et al.), urinary system (Chen et al.), and digestive system (Bein et al.; Charrez et al.). Lee-Montiel *et al.*, merged stem cell biology with tissue microengineering to generate liver and cardiac MPSs derived from the same induced pluripotent stem cell (hiPSC) line, and then applied their integrated multi-Organ Chips platform to study drug-drug interactions (Lee-Montiel et al.). Charrez *et al.*, developed a MPS populated with beating cardiomyocytes that were differentiated from iPSCs. The authors used this biodevice to evaluate cardiac safety of candidate therapeutics. Specifically, they studied the impact of repurposed drugs, hydroxychloroquine (HCQ) or azithromycin (AZM), for treating severe acute respiratory syndrome coronavirus 2 (SARS-CoV-2). They found that exposure to AZM lead to arrhythmias, and treatment with HCQ and AZM combination therapy synergistically increased QT interval—a measurement of heart ventricles depolarization and repolarization (Charrez et al.), highlighting the utility of MPS for cardiovascular safety pharmacology. Chen and colleagues in a Mini Review discuss emerging organoid and stem cell-derived microfluidic MPS kidney models, and their utility for studying renal disorders and drug-induced nephrotoxicity (Chen et al.). Cherne *et al.*, refined and applied their previously reported gut organoid flow chip (GOFlowChip) to create a dynamic and multicellular MPS that emulates dendritic cell (DC)-epithelial interactions in human stomach (Cherne et al.). The authors recreated DC chemotaxis through a synthetic hydrogel to demonstrate utility of their platform for real-time imaging of cell-cell interactions, and analyses of gastric response to challenge with pathogens, candidate drugs and mucosal vaccines. Bein *et al.*, used a human Intestine-on-a-Chip microfluidic device populated with intestinal epithelial and vascular endothelial cells to study host cellular and inflammatory responses following infection with a seasonal coronavirus (CoV) strain NL63 (Bein et al.). The authors observed that cell culture under microfluidic flow in the presence of peristalsis-like biomechanical forces led to angiotensin-converting enzyme 2 (ACE2) expression—the virus entry receptor for CoV NL63. Additionally, they found that treatment of CoV-infected intestinal chips with nafamostat mesylate, a synthetic pan-serine protease inhibitor that has been clinically approved in Japan and South Korea for over 3 decades, inhibited viral entry and resulted in a reduction in both viral load and pro-inflammatory cytokine secretion. This finding is in line with other reports of anti-corona-viral properties of nafamostat in other organ systems *in vitro* and *in vivo* (Yamamoto et al., 2020; Li et al., 2021; Niemeyer and Benam, 2021; Niemeyer et al., 2021; Takahashi et al., 2021).

To conclude, this research topic highlights several innovative, inter-disciplinary, translational efforts in development and application of human-relevant MPS that can advance predictive pharmacology and potentially reduce attrition rates in pharmaceutical developments. We hope this snapshot will encourage further support and enthusiasm for such studies and evolution of MPS technologies, so they become better characterized, pharmacologically benchmarked, and more widely accessible. This would in turn enable us collectively to address pressing unmet medical needs, alleviate the cost and shorten the duration of preclinical drug development processes, and reduce animal use in preclinical development and discovery biology.
